# Identification of the CRE-1 Cellulolytic Regulon in *Neurospora crassa*

**DOI:** 10.1371/journal.pone.0025654

**Published:** 2011-09-29

**Authors:** Jianping Sun, N. Louise Glass

**Affiliations:** Department of Plant and Microbial Biology, University of California, Berkeley, California, United States of America; Institute of Developmental Biology and Cancer Research, France

## Abstract

**Background:**

In filamentous ascomycete fungi, the utilization of alternate carbon sources is influenced by the zinc finger transcription factor CreA/CRE-1, which encodes a carbon catabolite repressor protein homologous to Mig1 from *Saccharomyces cerevisiae.* In *Neurospora crassa*, deletion of *cre-1* results in increased secretion of amylase and β-galactosidase.

**Methodology/Principal Findings:**

Here we show that a strain carrying a deletion of *cre-1* has increased cellulolytic activity and increased expression of cellulolytic genes during growth on crystalline cellulose (Avicel). Constitutive expression of *cre-1* complements the phenotype of a *N. crassa* Δ*cre-1* strain grown on Avicel, and also results in stronger repression of cellulolytic protein secretion and enzyme activity. We determined the CRE-1 regulon by investigating the secretome and transcriptome of a Δ*cre-1* strain as compared to wild type when grown on Avicel versus minimal medium. Chromatin immunoprecipitation-PCR of putative target genes showed that CRE-1 binds to only some adjacent 5′-SYGGRG-3′ motifs, consistent with previous findings in other fungi, and suggests that unidentified additional regulatory factors affect CRE-1 binding to promoter regions. Characterization of 30 mutants containing deletions in genes whose expression level increased in a Δ*cre-1* strain under cellulolytic conditions identified novel genes that affect cellulase activity and protein secretion.

**Conclusions/Significance:**

Our data provide comprehensive information on the CRE-1 regulon in *N. crassa* and contribute to deciphering the global role of carbon catabolite repression in filamentous ascomycete fungi during plant cell wall deconstruction.

## Introduction

Many microorganisms, especially filamentous fungi, secrete hydrolytic enzymes that play a key role in the degradation of plant cell wall polymers [1], [2], which consist mainly of cellulose, hemicellulose, and lignin. Plant cell wall degrading enzymes from filamentous fungi are currently being produced to aid in the development of sustainable and affordable biofuels from lignocellulosic material. In filamentous fungi, genes encoding hydrolytic enzymes involved in plant cell wall deconstruction are repressed during growth on easily metabolizable carbon sources, such as glucose. Carbon catabolite repression (CCR) is an important mechanism to repress the production of plant cell wall degrading enzymes during growth on preferred carbon sources. In addition to regulation by CCR, production of hydrolytic enzymes associated with plant cell wall degradation is induced to high levels only in the presence of plant cell wall biopolymers or their derivatives. Although some aspects of CCR that affect production of hydrolytic enzymes have been evaluated in the industrial species, such as *Hypocrea jecorina* (*Trichoderma reesei*) and *Aspergilli* (reviewed in [3], [4], [5], [6], a systematic analysis of CCR during plant cell wall degradation has not been performed for any filamentous fungus. Thus, we chose to evaluate CCR in the plant cell wall degrading filamentous fungus, *Neurospora crassa*, which has many experimental tools available that allow a system biology approach, including a near full genome deletion strain set and full genome oligonucleotide arrays for expression analysis [7]. Our ultimate goal is to understand, at a systems biology level, how a filamentous fungus alters both its internal and external metabolism in response to exposure to plant cell wall material.

Some aspects of CCR are mediated by Mig1/CreA/CRE1, a zinc-finger transcription factor conserved in most fungal species [8], [9], [10], [11], [12]. In *Saccharomyces cerevisiae*, under conditions of glucose sufficiency, Mig1 functions to repress transcription of ∼90 genes associated with utilization of alternative carbons sources, such as maltose, galactose and sucrose [13]. In *Aspergilli* and *H. jecorina*, CreA/CRE1 was shown to directly regulate genes involved in xylose, xylan, arabinose, proline and ethanol utilization, as well as penicillin biosynthesis (reviewed in [3], [4], [6]); the binding motif of CreA was shown to be 5′-SYGGRG-3′ and is context dependent [14], [15]. CreA is believed to regulate the transcription of genes in a “double-lock” manner [16], [17], [18], [19]. For example in *A. nidulans*, the genes of the ethanol regulon comprise the transacting regulatory gene *alcR* [20] and at least two main structural genes *alcA* (alcohol dehydrogenase I) [21] and *aldA* (aldehyde dehydrogenase) [22]. CreA directly represses the transcription of *alcR* as well as repressing *alcA* and *aldA* by competing with AlcR binding to promoter sequences [16], [18], [23]. Similarly, CreA also represses the xylanolytic system via direct repression of the pathway specific regulator, *xlnR* and both direct and indirect regulation of the structural gene *xlnA* [17], [19], [24]. In *H. jecorina*, CRE1 has been shown to bind to the promoter of cellobiohydrolase 1 (*cbh1*) which encodes one of the major hydrolytic enzymes involved in cellulose degradation [25] as well as the promoter of a gene encoding xylanase (*xyn1*) [26]. These data suggest that CRE1 may play a direct role in the regulation of the production of many plant cell wall degrading enzymes.

Although CCR has been studied in filamentous fungi, only a limited number of genes/systems have been clearly shown to be subject to direct CreA/CRE1 repression. Previously, we showed that *N. crassa* has a robust cellulolytic response to growth on plant cell walls and crystalline cellulose (Avicel), including induction and secretion of a large number of cellulases and hemicellulases [27]. Although deletion of *cre-1* in *N. crassa* was shown to increase the expression of invertase and increase amylase and β-galactosidase secretion [28], its effect on expression and/or secretion of cellulolytic enzymes has not been evaluated. In this study, we show that deletion of *cre-1* caused sustained expression of cellulase genes, resulting in higher cellulolytic enzyme activity. The repression of cellulolytic genes during growth on Avicel was correlated with *cre-1* transcription levels. Using full genome oligonucleotide arrays, we performed transcriptional profiling analyses to define the CRE-1 regulon and identified genes directly regulated by CRE-1 by chromatin-immunoprecipitation. By utilizing the near full genome deletion set developed for *N. crassa* [7], we identified novel genes in the CRE-1 regulon that, when mutated, have large effects on cellulolytic activity.

## Results

### Deletion of *cre-1* increased cellulolytic enzyme production

**Figure 1:**
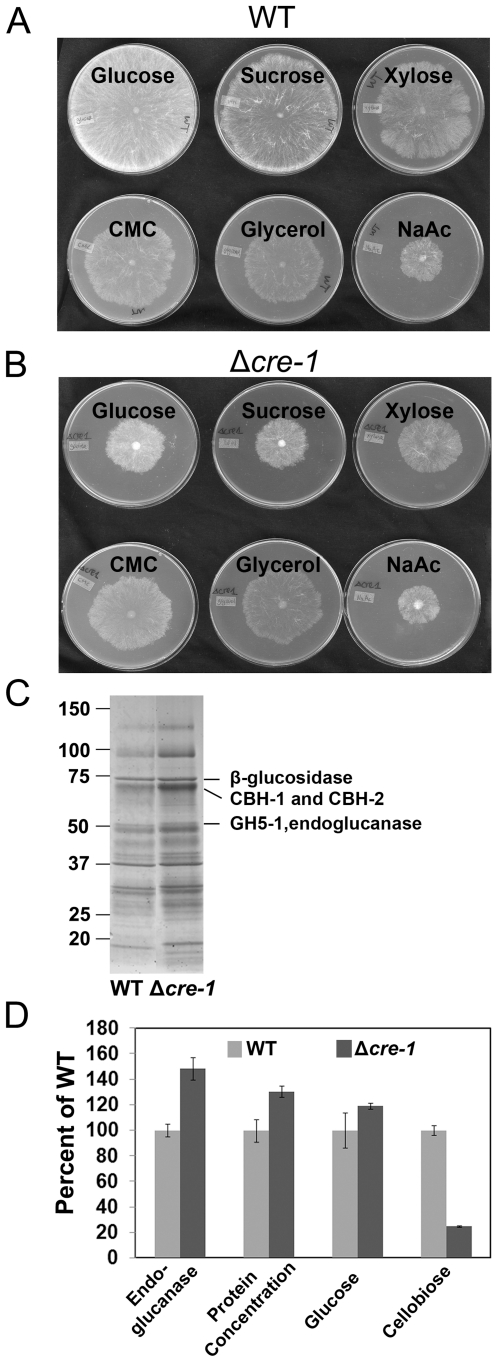
Phenotype of WT and Δ*cre-1* strains. Wild type (FGSC 2489) (A) and Δ*cre-1* (FGSC 10372) (B) strains were grown on different carbon sources at 30°C for 24 hrs. The carbon source in the media is indicated above each plate and all plates contained Vogel's salts and 2% of various carbon sources. C) SDS-PAGE of secreted proteins in culture filtrates from WT and Δ*cre-1* strains grown on Avicel for 7 days. Protein bands representing β-glucosidase (NCU04952), cellobiohydrolase 1 (NCU07340) and 2 (NCU09680), and endoglucanase 2 (NCU00762) are marked. D) Comparison of endoglucanase activity on azo-CMC, protein concentration, glucose and cellobiose concentration from Avicelase assays of 7-day culture supernatants from WT and Δ*cre-1* strains.

In *N. crassa*, the Δ*cre-1* mutant grows slower and denser than wildtype (WT) when grown on preferred carbon sources, such as glucose, sucrose or xylose [28], similar to the phenotype of *A. niger* and *T. reesei creA/cre1* mutants [11], [29], [30] (Figure 1A). However, no differences in growth rate or morphology from a WT strain were observed when Δ*cre-1* was grown on carboxymethylcellulose (CMC), glycerol or sodium acetate (NaAc) media (Figure 1B; Figure S1). When grown on 2% Avicel medium as a sole carbon source, the Δ*cre-1* strain consumed Avicel faster than WT (*e.g.* 3–4 days vs 5–6 days), secreted 30% more extracellular protein and showed 50% higher endoglucanase activity (Figure 1C and D). An aggregate Avicelase assay [27] (which measures combined β-glucosidase, endo-, and exo-cellulase activity) showed 20% higher glucose concentrations in the Δ*cre-1* strain as compared to WT (Figure 1D). However, less cellobiose was detected, suggesting increased secretion of β-glucosidase (which converts cellobiose into glucose; Figure 1C) in the Δ*cre-1* strain.

### Over-expression of *cre-1* increased carbon catabolite repression (CCR)

**Figure 2:**
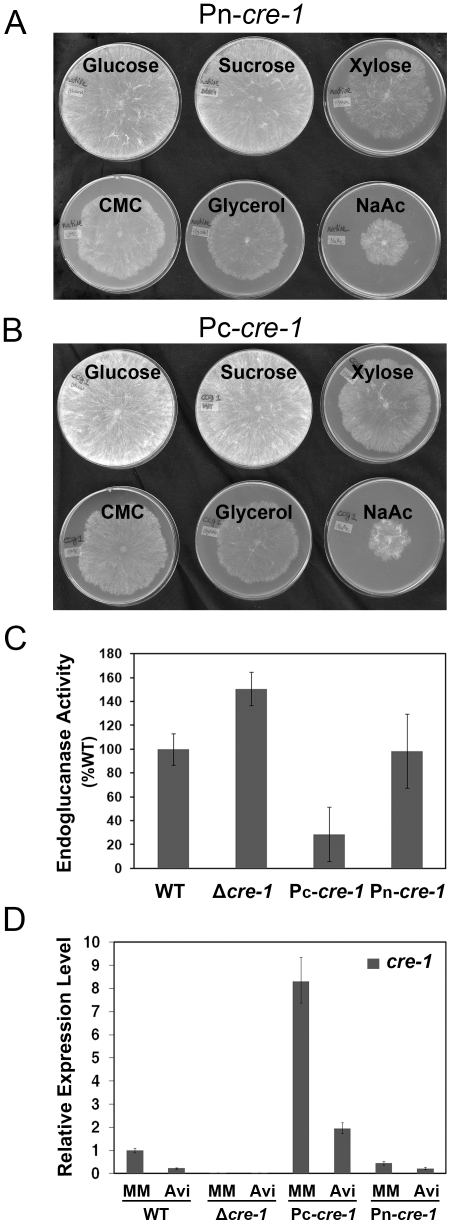
Complementation and gene expression of *cre-1*. A) Pn-*cre-1* (*his-3*::*Pnative-cre-1-gfp*; Δ*cre-1*) and B) Pc-*cre-1* (*his-3*::*Pccg-1-cre-1-gfp*; Δ*cre-1*) strains were grown on different carbon sources at 30°C for 24 hrs. The carbon source in the media is indicated above each plate and all plates contained 1× Vogel's salts and 2% of different carbon sources. C) Comparison of the azo-CMC activity of culture supernatants from WT, Δ*cre-1*, Pc-*cre-1* and Pn-*cre-1* strains grown on Avicel for 7 days. D) Quantitative RT-PCR of *cre-1* expression levels in WT, Δ*cre-1*, Pc-*cre-1* and Pn-*cre-1* strains. All strains were grown in MM for 16 hrs, and then transferred into sucrose or Avicel for an additional 4 hrs.

To determine whether *cre-1* expression levels affect cellulolytic activity in *N. crassa*, we constructed two strains with a C-terminal GFP-tagged CRE-1 in the Δ*cre-1* strain under the regulation of either the native *cre-1* promoter (strain Pn-*cre-1*) or the *ccg-1* promoter (strain Pc-*cre-1*) [31], [32]. Both Pn-*cre-1* and Pc-*cre-1* strains complemented the Δ*cre-1* phenotype (Figure 2A and B) and grew similarly to WT, although the Pc-*cre-1* strain showed slightly faster growth (an increase of ∼1 cm/day) under glucose/sucrose conditions. We observed a decrease in *cre-1* expression level for both WT and Pn-*cre-1* strains on Avicel as compared to sucrose by qRT-PCR (Figure 2D). However, *cre-1* expression levels in the Pc-*cre-1* strain were significantly higher than WT when grown on sucrose (8-fold) and showed an even higher expression level when grown on Avicel (10-fold higher) (Figure 2D). To determine whether increased levels of *cre-1* affected cellulolytic activity, we evaluated growth and secreted protein levels in WT, Pn-*cre-1* and Pc-*cre-1* when grown in Avicel. The Pn-*cre-1* strain had secreted protein levels and endoglucanase activity that were similar to WT (Figure 2C). However, the Pc-*cre-1* strain showed significantly lower secreted protein levels and endoglucanase activity (29% of WT). Undigested Avicel was present in growth medium in the Pc-*cre-1* strain at a time point where all of the substrate had been utilized in the Δ*cre-1*, WT and Pn-*cre-1* strains (data not shown). These data indicate that CCR in *N. crassa* was responsive to changes in *cre-1* expression level, similar to findings with *creA* in *A. nidulans* [33].

**Figure 3:**
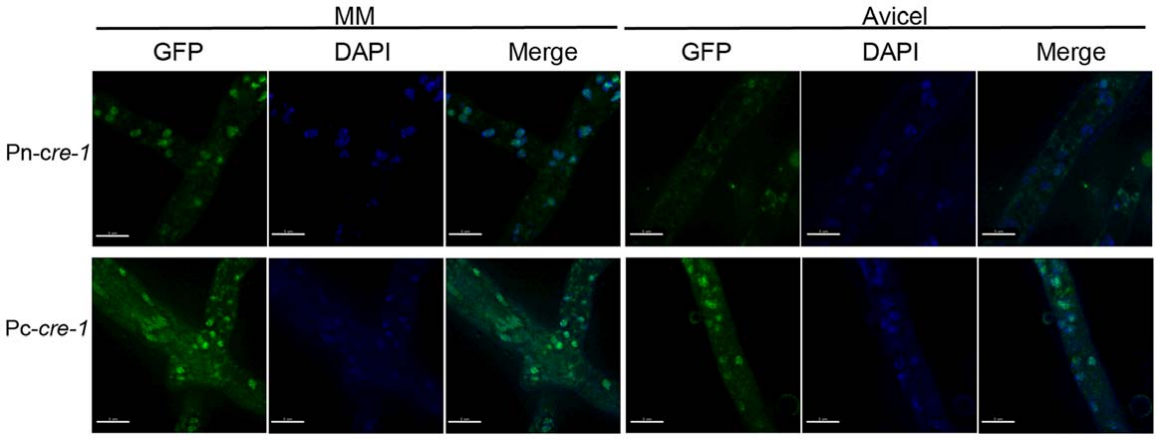
Subcellular localization of CRE-1-GFP. Strains with *cre-1* under control of the native promoter (Pn-*cre-1*) or the *ccg-1* promoter (Pc-*cre-1*) were grown on plates or in liquid MM for 16 hrs then transferred onto plates with Vogel's salts and either 2% sucrose (MM) or 2% Avicel a sole carbon source and allowed to grow for an additional 5–6 hrs (25°C). Fluorescence was evaluated using a Deltavision Spectris DV4 deconvolution microscope. Nuclei were also stained by DAPI. Scale bar = 10 µm.

In *S. cerevisiae*, the subcellular localization of Mig1 is regulated by glucose concentration [34]. Similarly, CRE1 in *Fusarium oxysporum* showed cytoplasmic localization under 0.01% glucose, but localized to nuclei when grown on 2% glucose or ethanol [35]. However, in *A. nidulans* and *Penicillium canescens*, GFP-tagged CreA did not show differential localization when exposed to various carbon sources [36], [37]. We evaluated the localization of CRE-1 when grown on agarose lacking any carbon source, on 2% sucrose and on 2% Avicel by performing live cell imaging of GFP tagged CRE-1. In cells grown in sucrose, CRE-1-GFP localized to nuclei, as expected for a glucose-dependent transcriptional repressor (Figure 3). However, in cultures grown on either agarose or Avicel, CRE-1-GFP also localized to nuclei in both the Pn-*cre-1* and Pc-*cre-1* strains (Figure 3). These data indicate that in *N. crassa* cellular localization of CRE-1 in response to carbon source does not play a major regulatory role for CCR.

### Increased expression level of cellulolytic enzymes was correlated with increased enzymatic activity in a Δ*cre-1* strain

**Figure 4:**
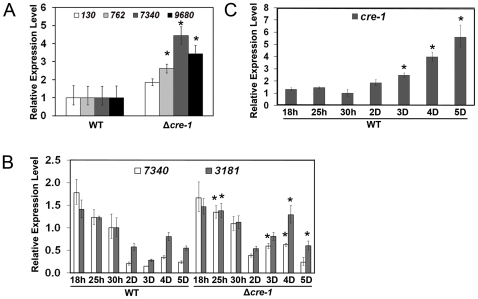
Gene expression patterns in wild type and Δ*cre-1* strains. A) Gene expression levels of *cbh-1* (NCU07340), *cbh-2* (NCU09680), *gh5-1* (NCU00762) and β-glucosidase (NCU00130) in WT (FGSC 2489) and Δ*cre-1* (FGSC 10372) strains. Expression levels for all genes were normalized to 1 in WT. Strains were grown in MM for 16 hrs followed by 4 hrs growth on Avicel. B) Gene expression levels of *cbh-1* (NCU07340) and putative CRE-1 target, NCU03181, in WT and Δ*cre-1* strains. Cultures were inoculated with conidia and harvested at time points shown post-inoculation. C) Gene expression levels of *cre-1* under identical conditions to that shown in (B). *actin* (NCU04173) gene expression levels were used as an endogenous control in all samples. Each reaction was done by triplicate. *P = 0.05.

To determine whether increased protein secretion and cellulase/endoglucanase activity in the Δ*cre-1* strain was due to a higher expression level of cellulolytic genes, we performed qRT-PCR of the major cellulase genes. As predicted, the expression levels of *cbh-1* (NCU07340), *cbh-2* (NCU09680) and endoglucanase-2 (NCU00762) were significantly higher in the Δ*cre-1* mutant as compared to WT when a 16-hr minimal media (MM) culture was shifted to Avicel for 4 hrs (Figure 4A). To assess expression of cellulases over time, we inoculated asexual spores (conidia) from either WT or the Δ*cre-1* mutant directly into Avicel and assessed expression of *cbh-1* and NCU03181 (encoding a conserved hypothetical protein; both *cbh-1* and NCU03181 are direct targets of CRE-1, see below). At the earliest time point (18 hr; germination of conidia is delayed on Avicel compared to MM [27]), the expression level for both *cbh-1* and NCU03181 was similar between WT and the Δ*cre-1* mutant (Figure 4B); expression levels of *cbh-1* and NCU03181 decreased at later time points. However, in WT, expression levels for *cbh-1* and NCU03181 were consistently lower than in the Δ*cre-1* strain at later time points (Figure 4B). We monitored *cre-1* expression during identical time points and observed that the expression of *cre-1* increased significantly after 2 days of growth on Avicel, up to a ∼6-fold increase after 5 days (Figure 4C). Thus, an increase in *cre-1* expression levels was correlated with reduced expression of predicted targets of CRE-1 (Figure 4B). It is possible that at later time points Avicel hydrolysis occurs at a rate such that glucose accumulates and triggers *cre-1* expression. However, the glucose concentrations at these later time points were undetectable by HPLC (data not shown). These observations suggest that the increase in cellulolytic activity of the Δ*cre-1* mutant is due to an inability to establish repression of cellulolytic genes once growth on cellulose has been established.

### Secretome comparison between Δ*cre-1* and WT strains

Analysis of the supernatant from a Δ*cre-1* culture grown on Avicel showed increased secretion of proteins, with a very similar pattern to WT (Figure 1C). To assess differences more accurately between proteins secreted in WT versus the Δ*cre-1* mutant, we analyzed the secretome using a shotgun proteomics approach. Supernatant from a 7-day old Δ*cre-1* culture grown on Avicel was digested with trypsin and analyzed by liquid chromatography nano-electrospray ionization tandem mass spectrometry (MS), as described [27]. A total of 31 proteins was detected in the Δ*cre-1* Avicel culture (Table S1), 30 of which were predicted to be secreted based on SignalP computational analysis (http://www.cbs.dtu.dk/services/SignalP/). The dataset included 9 of the 23 predicted cellulases and 5 of the 19 predicted hemicellulases in *N. crassa* genome [27], [38]. There were also 10 proteins with predicted activity on carbohydrates, 4 conserved hypothetical proteins, and 3 proteins with functions in other pathways (NCU07200, NCU08785 and NCU09518).

When compared with the secretome of a WT strain grown on Avicel and *Miscanthus* [27], 26 proteins overlapped with that of the Δ*cre-1* strain (Table S1), which included all 9 cellulases, 5 hemicellulases, 3 conserved hypothetical proteins, and others with predicted activity on carbohydrates. Five proteins were *cre-1* specific, 3 of which have predicted activity on carbohydrates, including NCU05598 (rhamnogalacturonase), NCU09664 (acetylxylan esterase) and NCU09518 (glucooligosaccharide oxidase), plus two additional proteins (NCU00449 (conserved secreted protein) and NCU07200 (metalloprotease)).

### Identification of the CRE-1 regulon

**Figure 5:**
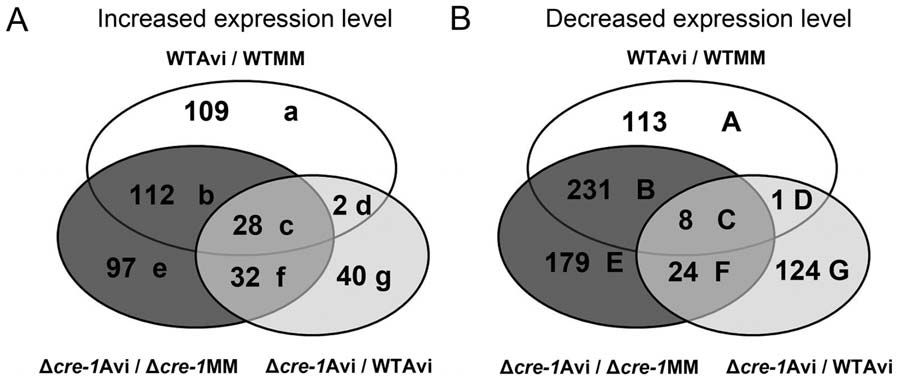
Venn diagram of the transcriptome of wild type and Δ*cre-1* strains. A) Overlap among genes that exhibit statistically significant increased expression level in Δ*cre-1* strain relative to the WT strain (see Table 1 for details). There are a total of 271 genes that showed increased expression in the Δ*cre-1* strain (for FunCat analysis, see Figure 7). Data sets marked as c, d, e, f and g were under Avicel growth conditions, with an additional 75 genes that showed increased expression levels in the Δ*cre-1* strain in MM compared to WT (Table 1). B) Overlap among genes that showed a statistically significant decrease in expression level in the Δ*cre-1* strain relative to WT. This set includes 381 genes identified from Avicel cultures marked as C, D, E, F and G, plus 80 genes that showed decreased relative expression levels in a Δ*cre-1* compared to WT when grown on MM (Table 1; for FunCat analysis, see Figure 7).

As a complement to the secretome analysis, we performed transcriptional profiling of WT and Δ*cre-1* strains grown on sucrose versus on Avicel (Figure 5; Figure S2). Preliminary experiments indicated that exposure of a 16 hr MM culture to Avicel for 4 hrs was sufficient to induce gene expression of cellulase/hemicellulase genes (Figure 4A; Figure S3). We assessed expression levels of a 16 hr culture of Δ*cre-1* after a switch for 4 hrs to either MM (control) or Avicel and compared them to expression levels of 16-hr WT mycelia switched to MM or Avicel for 4 hrs. Among the 10,910 70-mers representing predicted *N. crassa* genes, relative expression levels for 6,614 genes were detected (Table S2, p. 1). As expected, expression levels obtained by microarray analysis mirrored that of quantitative RT-PCR analysis of expression levels for a set of cellulase and hemicellulase genes (Table S2; Figure S3).

**Table 1:** Summary of transcriptional profiling results.

	Number of genes with indicated change				
Type of change in	Amt of change	Δ*cre-1* Avi	Δ*cre-1* Avi	Δ*cre-1* MM	WT Avi
gene expression	(n-fold)	vs WT Avi	vs Δ*cre-1* MM	vs WT MM	vs WT MM
	>10	2	57	8	34
Increase	>5	19	94	14	70
	>2	102^a^	269^a^	75^a^	251
	>10	4	35	none	17
Decrease	>5	18	101	7	94
	>2	157^b^	442^b^	80^b^	353

a represents genes that showed an increased expression level in a Δ*cre-1* mutant.

b represents genes that showed a decreased expression level in a Δ*cre-1* mutant.

**Figure 6:**
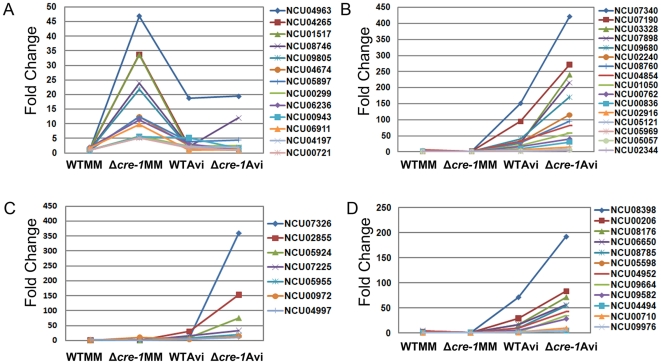
Relative expression levels of *N. crassa* genes predicted to be regulated by CRE-1. A) Genes with increased expression level in the Δ*cre-1* mutant as compared to WT under MM culture conditions; expression levels of these genes are shown for cultures transferred to MM or Avicel for 4 hours. NCU00721 (proline permease) and NCU09805 (α-amylase A) have been identified as direct targets of CreA in *Aspergilli* [15], [40]. B) Expression level of 16 of the 23 predicted cellulases in WT or the Δ*cre-1* mutant from cultures transferred to MM or Avicel for 4 hours. NCU07340 (*cbh-1*) is a direct target of CRE1 in *H. jecorina* [25]. C) Expression levels of 7 of the 19 predicted hemicellulases in WT or the Δ*cre-1* mutant from cultures transferred to MM or Avicel for 4 hrs. NCU02855 is a known target of CRE-1 in other systems [48]. D) Expression levels of genes with functions associated with plant cell wall degradation that showed increased expression in the Δ*cre-1* mutant versus WT following transfer to either MM or Avicel for 4 hrs.

An important role of CRE-1 is its repression of genes encoding enzymes involved in the utilization of alternative carbon sources. In cultures grown in MM, 75 genes showed increased relative expression levels in the Δ*cre-1* mutant versus WT (Table 1; Table S2, p. 2). Of these 75 genes, five showed a greater than 20-fold increase in expression level in the Δ*cre-1* mutant under MM conditions, including one direct target of CRE-1 identified in other systems, NCU09805 (α-amylase A) [39], [40], a predicted target of CreA in *Aspergilli* (glucoamylase; NCU01517) [39], a high affinity glucose transporter (NCU04963), a protein related to β-fructofuranosidase (NCU04265), and a starch binding protein (NCU08746) (Figure 6A). Ten other genes had a >5-fold increase in Δ*cre-1* strain under MM conditions, including one direct target of CreA in *A. nidulans*, *prnB* (proline-specific permease; NCU00721) [15]. The remaining genes included an additional transporter gene, NCU05897 (glucose/galactose transporter), NCU00943 (trehalase), 4 genes with functions associated with sugar or fatty acid metabolism, and 3 genes encoding hypothetical proteins. The remaining 60-gene set included two additional direct targets of CRE-1 identified from other systems, NCU01754 (*A. nidulans* alcohol dehydrogenase I) [18] and NCU02184 (*Trichoderma harzianum* endochitinase 2) [41] and one predicted target, NCU07788 (related to transcription activator *amyR* in *A. nidulans*) [42]. Five additional genes in this set encoded predicted sugar transporters, (NCU01633, NCU04537, NCU10021, NCU00821, and NCU05627), one encoded a nucleoside transporter (NCU08148), several genes had annotation suggesting a role in alternative carbon source utilization (e.g. pyruvic acid), and 26 genes encoded putative or hypothetical proteins. No cellulolytic genes were induced in the Δ*cre-1* mutant when grown in MM, consistent with the requirement for induction, as well as relief from CCR.

A recently published paper reported that 207 genes in *H. jecorina* (*T. reesei*) were differentially regulated in a Δ*cre1* versus a wild-type strain during glucose assimilation under chemostat-type continuous cultivation [30]. Of these, a 118-gene set was predicted to be repressed by CRE1, which was enriched for hypothetical and transport proteins, while a 72-gene set was predicted to be induced. We compared the 118 and 72 gene sets (total of 190 genes) with the *N. crassa* genome and identified 103 orthologous genes. Of these, 6 genes showed increased expression levels in the *N. crassa* Δ*cre-1* mutant under MM conditions, similar to *H. jecorina* (Table S3), while 2 genes that showed increased expression level in *H. jecorina* Δ*cre1* mutant, but showed decreased expression levels in *N. crassa*. Meanwhile, 10 genes that showed decreased expression levels in the *H. jecorina* Δ*cre1* mutant also showed decreased expression levels in the *N. crassa* Δ*cre-1* mutant (Table S3). Of the 18 genes that overlap between the *H. jecorina* and *N. crassa* datasets, five encode proteins with transport functions (Table S3).

Under Avicel growth conditions, we identified 102 genes that showed a >2-fold increase above WT in the Δ*cre-1* mutant (Figure 5A; c, d, f and g; Table S2, p. 3). FunCat analysis [43] of these 102 genes showed that only 3 major categories of genes were enriched (P< 10e-5): genes related C-compound and carbohydrate metabolism, protein synthesis and protein with binding function or co-factor requirement. Genes enriched in C-compound and carbohydrate metabolism category included 16 of the 23 predicted cellulase genes (Figure 6B). Most of these 16 cellulase genes showed a 2–3 fold increase in expression in the Δ*cre-1* mutant. However, gene expression levels for two GH61 proteins (*gh61-6*; NCU03328 and *gh61-4*; NCU07898) and one GH45 protein (*gh45-1*; NCU05121) increased over 6-fold. The gene encoding *cbh-1* (NCU07340) showed the highest expression levels in Avicel in both WT and the Δ*cre-1* mutant, with expression increasing almost 3-fold in the Δ*cre-1* mutant (Figure 6B).

Seven of the 19 predicted hemicellulase genes also increased in expression level at least 2-fold in Δ*cre-1* mutant (Figure 6C), with one gene, NCU07326, encoding a GH43 enzyme (predicted arabinofuranosidase), increasing 28-fold in relative expression level. Expression levels of two additional genes, NCU02855 (*gh11-1*) and NCU05924 (*gh10-1*) showed a more than 5-fold increase in the Δ*cre-1* strain as compared with WT when grown in Avicel. By contrast, two predicted hemicellulase genes (NCU08189; *gh10-2*), NCU02343 (alpha-L-arabinofuranosidase A) showed a ∼3–5 fold decrease in expression under Avicel conditions in the Δ*cre-1* mutant as compared to WT. An additional 12-gene set showed increased expression level in the Δ*cre-1* mutant, were predicted to be secreted and had annotations associated with a role in plant cell wall degradation (Figure 6D). Most of these genes showed a 2-6-fold increase in expression level in the Δ*cre-1* mutant. However, one gene, NCU09664, encoding a probable acetyl xylan esterase, increased in expression 17-fold in the Δ*cre-1* mutant. Other genes included NCU08398 (aldose epimerase), NCU00206 (cellobiose dehydrogenase), NCU08176 (pectate lyase), NCU06650 (phospholipase A2) and NCU04952 (β-glucosidase).

**Figure 7:**
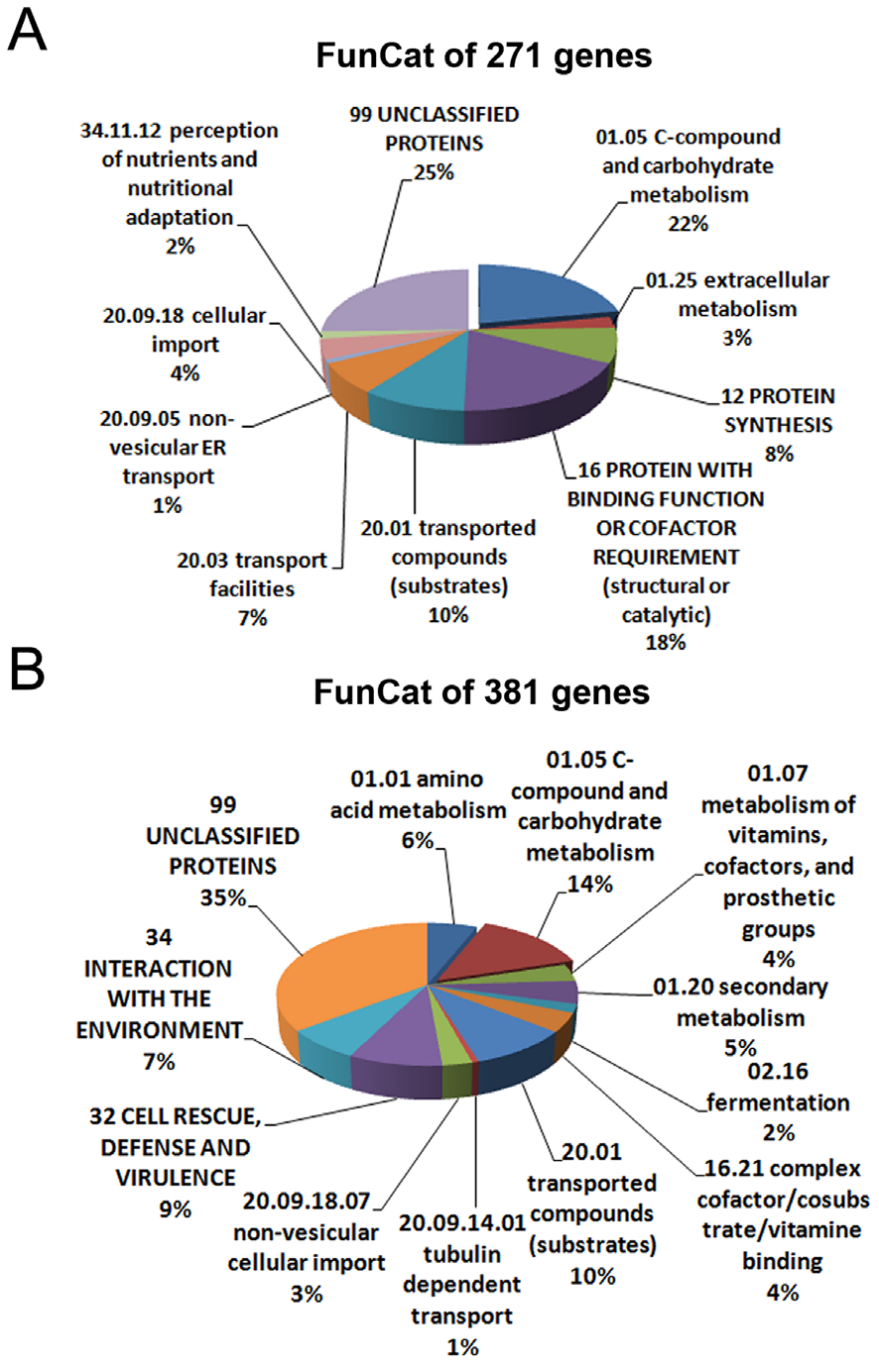
Functional category (FunCat) analysis of potential CRE-1 target genes. A) Functional category (FunCat) analysis of the 271 genes that showed increased expression levels in the Δ*cre-1* mutant relative to WT (CRE-1 repressed genes). There were 77 genes classified into the category of C-compound and Carbohydrate metabolism, which included most of the predicted cellulase and hemicellulase genes. B) FunCat analysis of 381 genes that showed a decrease in relative expression level in the Δ*cre-1* mutant as compared to WT (CRE-1 activated genes). Of these, 65 genes were in the C-compound and Carbohydrate metabolism functional category.

A comparison of the Δ*cre-1* expression profile on Avicel versus MM showed that 97 genes (Figure 5A; e) had a *cre-1*-specific increase. Functional category analysis [43] of the entire Δ*cre-1* dataset of 271 genes (102+97+75; 3 gene overlap) showed a significant enrichment in the functional categories of C-compound/carbohydrate metabolism (P = 7.56e-26), extracellular metabolism (P = 8.30e-7), protein with binding function or cofactor requirement (P = 9.22e-06), C-compound/carbohydrate transport (P = 5.32e-06), transport facilities (P = 5.54e-07) and protein synthesis (P = 1.86e-09) (Figure 7A, Table S2, p. 4). In addition, a large number genes encoding unclassified proteins was also within this dataset (25%).

Most reports characterizing the function of *cre-1* focus on the repression of genes involved in utilizing alternative carbon sources. However, it is also possible that CRE-1 plays a role in gene activation. For example, Mig1 in *S. cerevisiae* has been suggested to function as a transcriptional activator [13], [44]. Under Avicel conditions, a total of 336 genes were identified whose expression level decreased in the Δ*cre-1* mutant (Figure 5B; C, D, E, F and G) with 157 of these genes showing a greater than 2-fold lower expression level. Eighty genes showed a reduced expression level in Δ*cre-1* versus WT from MM cultures (Table 1). These two datasets (336+80) overlapped by 35 genes, giving a total of 381 genes that showed lower relative expression level in the Δ*cre-1* strain. Functional category analysis [43] of these 381 genes showed that 35% encoded proteins with unclassified functions, constituting the largest group (Figure 7B). The second largest category of 65 genes fell into the C-compound and carbohydrate metabolism category (P = 9.53e-11). Other enriched functional categories included genes related to amino acid metabolism (28 genes, P = 1.96e-05), genes involved in cell rescue, defense and virulence (P = 8.02e-05), as well as genes involved in interaction with the environment (P = 3.37e-04) (Figure 7B, Table S2, p. 5).

### Analysis of the CRE-1 binding motif and identification of direct targets by ChIP-PCR

By molecular and biochemical analyses, CreA in *A. nidulans* has been shown to bind adjacent 5′-SYGGRG-3′ motifs in the promoter regions of *ipnA* (penicillin biosynthesis) [14], *prnD* and *prnB* (proline utilization) [15], *alcR* and *alcA* (ethanol utilization) [16], [18] and *xynA* (endoxylanase) [19]. In *H. jecorina*, CRE1 binds to similar motifs in the promoter of *cbh1* and *xyn1* [26]. However, by MEME [45], MDscan [46] and BioProspector [47], we did not identify an enrichment for motifs resembling 5′-SYGGRG-3′ in an analysis of predicted CRE-1 target genes (the 271 or 381 gene sets; Figure 7A, B), as this motif is fairly common in the genome (3.14 motif/gene). However, CreA has been shown to bind to closely linked 5′-SYGGRG-3′ motifs [15], [18], [48]. We therefore used 5′-SYGGRG-3′ motif specifically to search the upstream 1 kbp promoter regions of a subset of CRE-1 putative targets (PATSER; http://rsat.bigre.ulb.ac.be/rsat), which included genes enriched in the C-compound and carbohydrate metabolism category (77 genes that increased in expression level and 65 genes that showed decreased expression in the Δ*cre-1* mutant), and specifically flagged cases with adjacent 5′-SYGGRG-3′ motifs. Promoter regions of 38 of the 77 gene set and 31 of the 65 gene set contained adjacent 5′-SYGGRG-3′ motifs, and many contained more than two adjacent motifs (Table S4). These putative CRE-1 target genes were either cellulolytic, carbon source utilization or sugar transporter genes.

**Figure 8:**
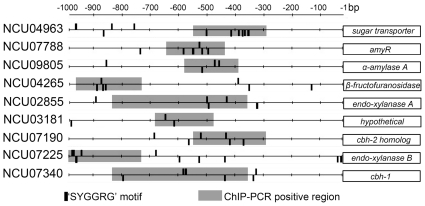
Schematic representation of 5′-SYGGRG-3′ motifs in promoter regions and regions enriched by ChIP-PCR. Vertical black bars indicate the location 5′-SYGGRG-3′ motifs in 1 kbp promoter regions of 9 putative CRE-1 target genes (NCU number on left and predicted function on right). Shaded box indicates region of promoters that showed enrichment via chromatin-immunoprecipitation-PCR experiments. See Tables S4, S5 and Figure S4 for further details.

To identify direct targets of CRE-1, we performed Chromatin immunoprecipitation (ChIP)-PCR using our Δ*cre-1* (*cre-1-gfp*) construct and anti-GFP antibodies for immunoprecipitation. Promoters of six genes that showed high expression level under sucrose conditions and 10 genes that showed high expression levels under cellulolytic conditions in the Δ*cre-1* mutant and that had multiple 5′-SYGGRG-3′ motifs in the promoter regions were chosen. Antibodies to Pol II and the promoter region of a constitutively expressed gene, glyceraldehyde-3-phosphate dehydrogenase (*gpd-1*; NCU01528) were used as a positive control (Figure S4). As a negative control, we used a 332 bp region in the *N. crassa* supercontig 10.7 (2726–3057) that showed no expression under any experimental conditions (unpublished observations) (Figure S4). Promoters of four of six genes that showed increased expression level in the Δ*cre-1* mutant under sucrose conditions were specifically enriched by ChIP-PCR, including one direct CreA target in *A. oryzae, amyA* (α-amylase A; NCU09805) [40], a major facilitator superfamily (MFS) monosaccharide transporter (NCU04963), a predicted β-fructofuranosidase (NCU04265) and *col-26* (NCU07788; related to the transcription factor AmyR, which is required for induction of amylolytic genes, such as *amyA* in *A. nidulans* [39]) (Figure 8; Figure S4 and Tables S4, S5). Promoters regions of NCU06911 (conserved hypothetical protein) and NCU08746 (glucoamylase) were not enriched. Genes that showed increased expression in the Δ*cre-1* mutant under cellulolytic conditions that were positive by ChIP-PCR included NCU07340 (*cbh-1*) and NCU02855 (*gh11-1*; predicted xylanase), both of which are direct CRE1 targets in *T. reesei* [25], [48]. In addition, NCU03181 (a hypothetical protein), NCU07190 (*gh6-3*; predicted endoglucanase) and NCU07225 (*gh11-2*; predicted xylanase) were also positive. Promoter regions of NCU08176 (probable pectate lyase) and NCU06509 (conserved hypothetical protein) were not enriched. ChIP-PCR results for promoter regions for three genes (NCU07326, NCU08760 and NCU00762) were equivocal (Table S5). Promoter regions bound by CRE-1-GFP contained closely spaced consensus 5′-SYGGRG-3′ motifs, although not all adjacent 5′-SYGGRG-3′ motifs were enriched by immunoprecipitation of CRE-1-GFP. For example, there are 2 regions (position −322 to −337; −570 to −583) in the promoter of *cbh-1* containing closely spaced consensus 5′-SYGGRG-3′ motifs. ChIP-PCR showed that in the *cbh-1* promoter, a DNA fragment containing one region (−570 to −583) was specifically enriched in the CRE-1-GFP pulldown, but not DNA fragments from the second region (−322 to −337) (Figure 8; Tables S4, S5). These data indicate, similar to studies in *Aspergilli* and *H. jecorina*, genes that contain multiple adjacent 5′-SYGGRG-3′ motifs in their promoter regions are more likely be the direct targets of CRE-1, but that other factors play a role in DNA binding rather than just consensus binding sites.

### Characterization of extracellular proteins and cellulase activity in strains containing deletions of genes within the CRE-1 regulon

Unlike other filamentous fungi, *N. crassa* has a near full genome deletion strain set [7], thus enabling the facile screening of mutants for phenotypes. Of the 102 genes identified within the CRE-1 regulon under cellulolytic conditions (Table 1), homokaryotic strains containing deletions of 43 genes were available. Thirteen of these 43 strains have been previously evaluated for cellulolytic capacity, including strains containing mutations in *cbh-1* (NCU07340), *cbh-2* (NCU09680), endoglucanase-2 (NCU00762; *gh5-1*) and β-glucosidase (NCU04952; *gh3-4*), all of which showed a cellulolytic phenotype [27]. The remaining 30 deletion strains were tested on media containing Avicel as a sole carbon source and assessed for total secreted protein and endoglucanase activity on azo-CMC as compared to the WT strain (Table S6). For the majority of these deletion strains, no significant difference in activity from the WT strain was observed. However, deletions in two genes, NCU06509 and NCU06704, showed a decrease in both protein secretion and enzyme activity. NCU06509 encodes a putative protein with a predicted transmembrane domain and is conserved in some fungi, such as in *Aspergillus sp*. NCU06704 encodes a homolog of *S. cerevisiae YSY6* and the mammalian protein RAMP4. Ysy6 suppresses secretion defects of an *Escherichia coli secY* mutant [49], while RAMP4 was identified in a biochemical search for proteins associated with the mammalian translocon [50].

**Figure 9:**
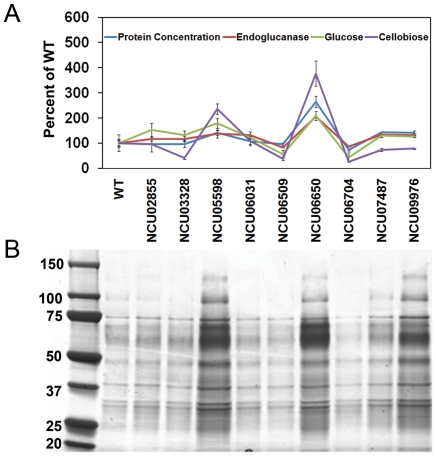
Enzyme activity and protein profile of culture supernatants from strains containing deletions of genes in the CRE-1 cellulolytic regulon. A) Total secreted protein, endoglucanase activity on azo-CMC, glucose and cellobiose concentration from 5-hr Avicelase assays of 7-day old culture supernatants from WT and deletion strains grown in Avicel as a sole carbon source. B) SDS-PAGE of proteins from unconcentrated culture supernatants of WT and deletion strains using same samples as from (A). Bars represent standard deviation.

Surprisingly, a strain containing a deletion of NCU06650, which is predicted to encode a homolog of a secreted prokaryotic phospholipase A2 (PLA2) resulted in significantly increased protein secretion, endoglucanase and Avicelase activity (Figure 9A, B). Three additional deletion strains (ΔNCU05598, ΔNCU07487 and ΔNCU09976) showed slightly higher levels of secreted proteins and increased cellulolytic enzyme activity (Figure 9A; Table S6). NCU07487 encodes a predicted periplasmic β-glucosidase (GH3), while NCU09976 encodes a probable intracellular rhamnogalacturonan acetyl esterase. The protein product of NCU05598, which encodes a predicted rhamnogalacturonase B, was detected by mass spectrometry specifically in the Δ*cre-1* mutant (Table S1). A homolog of NCU05598 in *Aspergillus aculeatus* has been shown to cleave pectin [51] and functions with rhamnogalacturonase A (RGX1). The hyper-secretion *T. reesei* RUT C30 strain also lacks *rgx1* [52]. Thus, the characterization of the CRE-1 cellulolytic regulon, which includes genes directly and indirectly regulated by CRE-1, identified genes that when mutated, resulted in strains showing a cellulolytic phenotype.

## Discussion

Carbon catabolite repression is ubiquitous among microbes and in eukaryotic species has been studied most extensively in *S. cerevisiae* where it is involved in repressing transcription of genes encoding enzymes for the utilization of maltose, sucrose and galactose [13], [53]; ∼90 genes are thought to be direct targets of Mig1 (*cre-1* homolog) [54], [55], [56]. In *S. cerevisiae*, Mig1 is phosphorylated by Snf1 kinase upon glucose depletion, resulting of exit of Mig1 from the nucleus [57]. Mig1 also recruits the global repressor complex, Cyc8-Tup1 to repress transcription [44]. In *T. reesei*, phosphorylation of Cre1 is required for DNA binding [58], although the Snf1 homolog in *T. reesei* apparently does not regulate CRE-1 [59]. In *S. cerevisiae*, hexokinase PII plays a role in glucose repression [60]. However, lack of hexokinase activity in an *A. nidulans frA1* mutant did not affect the glucose repression of enzymes involved in alcohol or L-arabinose catabolism [61]. These observations suggest divergence of regulatory CCR pathways in yeast versus filamentous fungi.

In this study, we used a systems biology approach employing expression profiling/mass spectrometry and mutant analyses to identify genes/proteins that are affected in expression level/cellulase activity in the *N. crassa* Δ*cre-1* mutant when grown in MM versus cellulose. We identified genes known to be directly regulated by CRE-1 homologs in other systems and also a large number of other genes of predicted or unknown function whose relative expression level increased substantially in the Δ*cre-1* mutant. These genes may be regulated directly or indirectly by CRE-1. By ChIP-PCR, we identified 9 direct CRE-1 targets in *N. crassa*, four regulated by CRE-1 under MM conditions and 5 regulated during growth on Avicel. A number of these direct CRE-1 targets are conserved among filamentous fungi, including *A. oryzae amyA* (NCU09805), which is involved in starch degradation [40]. The regulator of *amy A*, AmyR, is predicted to be regulated by CreA based on mutational analyses in *A. nidulans* [42]. Two additional *N. crassa* CRE-1 targets under cellulolytic conditions, *cbh-1* (NCU07340) and xylananse A (NCU02855) have been identified as CRE-1 targets in *H. jecorina* and/or *A. nidulans* [19], [25], [48]. The remaining six genes identified as CRE-1 targets in *N. crassa* have not previously been identified and included a hypothetical protein of unknown function (NCU03181), an additional xylanase (NCU07225) and *gh6-3* (NCU07190). Interestingly, a MFS monosaccharide transporter (NCU04963) was identified as a direct target of CRE-1. Similarly, the expression of another MFS transporter in *A. niger* (*mstA*) and a different MFS transporter in *A. nidulans* (*mstE*) have been shown to be affected by mutations in *creA* [62], [63], suggesting that CRE-1 may directly regulate genes involved in sugar transport, in addition to regulating genes encoding regulatory/enzymes associated with utilization of alternative carbon sources.

CRE-1 and its homologs have been shown to bind a 5′-SYGGRG-3′ consensus motif [14], [15], [64]. This motif is quite common in *N. crassa* promoter regions and there is no significant enrichment of this sequence in genes under regulation of CRE-1 as compared to the whole genome. In *Aspergilli* and *H. jecorina*, it has been proposed that this 5′- SYGGRG -3′ pattern is not specific enough to be predictive [30], [65]. CreA/CRE1 target promoters tend to contain multiple adjacent predicted CreA/CRE1 binding motifs. For example, the *A. nidulans alcR* promoter has nine consensus CreA binding sites, but only a pair of two adjacent sites were found to be functional *in vivo* [66]. Similarly, ten CRE1 binding sites are present in the promoter of *H. jecorina xyr1* [67]. CRE1 was shown in *H. jecorina* to bind to adjacent motifs in the promoter of *cbh1* [25], while a deletion in predicted adjacent CreA binding sites in the *A. niger aguA* promoter resulted in a significant increase in gene expression [68]. Target regions of promoters identified by ChIP-PCR in this study also contained adjacent 5′-SYGGRG-3′ motifs, consistent with previous report that CreA may function as a dimer [26]. However, it was not possible to predict whether a promoter would be bound by CRE-1 solely on the presence of adjacent 5′-SYGGRG-3′ motifs, suggesting that additional regulatory factors affect the specificity of CRE-1 binding.

In *S. cerevisiae*, a number of studies have shown that Mig1 may also function as an activator [44], [69]; a similar role has also been postulated for CreA/CRE1 in *A. nidulans* and *H. jecorina* [8], [9]. In *H. jecorina*, a number of genes showed decreased expression level in a Δ*cre1* mutant and were thus predicted to be positively regulated by CRE1 [30]. We also observed a large number of genes that showed decreased expression in the Δ*cre-1* mutant. In all filamentous fungi studied to date, loss-of-function mutations in *cre-1* homologs result in strains that show morphological defects when grown under rich carbon sources. In a *creA* mutant in *A. nidulans*, alterations in glycolytic enzyme activities, metabolite profile changes and depression of primary metabolism pathways occurs, indicating a perturbation of primary metabolism and associated co-factors [70], [71]. CRE-1 also interacts with pathways known to be important for growth and polarity. For example, mutations in the regulatory subunit (*mcb*) of cAMP protein kinase A (PKA), results in a *N. crassa* mutant that shows apolar growth and increased PKA activity [72]. By contrast, a Δ*cre-1* mutant shows reduced PKA activity and a *mcb*; Δ*cre-1* double mutant partially restores growth rate and hyphal polarity [28]. PKA also plays a role in the glucose response in both filamentous fungi and in yeast [13], [73]. A global analysis of CRE-1 binding across the genome (*e.g.* ChIP-seq) and under a variety of carbon source utilization scenarios will elucidate direct and indirect targets and will reveal additional information on the context of CRE-1 binding sites and potential activating and repressor functions for this important transcription factor.

From the transcriptome and secretome analyses of the Δ*cre-1* mutant when grown on crystalline cellulose, we identified many genes/proteins that were associated with cellulose degradation. Strains containing deletions in many of the genes within the CRE-1 cellulolytic regulon did not show a significant phenotype when grown on Avicel. However, a strain containing a deletion of NCU06650, encoding a predicted secreted phospholipase A2 (sPLA_2_), exhibited significantly increased protein secretion (especially of CBH-1 and CBH-2) and had increased cellulase activity. This result was unexpected, as expression of NCU06650 increased 3-fold in the Δ*cre-1* mutant. However, it is clear that compensatory mechanisms occur in strains with deletions of genes important for plant cell wall degradation, including increased protein secretion and cellulase activity [27]. sPLA_2_ has not previously been linked with response to cellulose, although the sPLA_2_ homolog in *A. oryzae* (*splaA*) is induced under carbon starvation. In *A. oryzae*, SplaA localizes to hyphal tips, is secreted and shows high enzyme activity towards phospholipid membranes and phosphatidylcholine [74]. sPLA_2_ was first identified in the mycorrhizal ascomycete species, *Tuber borchii* [75], where it was hypothesized to have a signaling and/or membrane re-modeling role associated with plant colonization. In mammalian cells, secreted phospholipases are associated with mobilization of fatty acids, including the signaling molecule arachidonic acid and are associated with allergic and systemic inflammatory/autoimmune diseases [76]. Further characterization of the ΔNCU06650 mutant and the sPLA_2_ protein in *N. crassa* will be informative as to its mode of action and how it affects secretion and cellulase activity.

In summary, our secretome/transcriptome analyses demonstrated that CRE-1 functions as a global transcription factor in *N. crassa* and affects both gene repression and activation, both directly and indirectly. In the presence of glucose, CRE-1 represses genes involved in alternative carbon source utilization, such as amylolytic and alcohol utilization genes. Several transporter genes repressed by CRE-1 under minimal media conditions suggests a role for these MFS proteins in transporting alternative sugars. Under cellulolytic conditions, CRE-1 regulates genes involved in plant cell wall utilization by directly binding to adjacent motifs in promoter regions and also may compete for binding with positive regulatory factors. For example, CRE-1 binds to the promoter region of *cbh-1* in *N. crassa* and may compete for binding with pathway specific cellulolytic regulator required for induction; the identity of cellulolytic regulators in *N. crassa* is currently unknown. Our data provides comprehensive information on the role of CRE-1 in the plant cell wall degradation regulon in *N. crassa*. The tools available in *N. crassa* [7] and the ease of genetic manipulation will facilitate the dissection of how *N. crassa*, a cellulolytic fungus, modifies it transcriptional, metabolic, secretory capacity and extracellular enzymes repertoire to efficiently digest its natural substrate, plant cell walls. The integration of CCR of genes encoding enzymes required for plant cell wall deconstruction versus positive regulators of genes involved in plant cell wall deconstruction will decode regulatory aspects of hydrolytic enzyme production.

## Methods

### Strains, growth techniques and microscopy

The *Neurospora crassa* wild type (WT) strain (FGSC 2489) and the *cre-1* gene deletion strain (Δ*cre-1*) (FGSC 10372) [28] were obtained from the Fungal Genetics Stock Center (FGSC) [77]. The (*his-3*; Δ*cre-1 a*) strain was obtained by crossing FGSC 6103 (*his-3 A*) with FGSC 10372. *N. crassa* was grown on Vogel's salts [78] with 2% (w/v) carbon source (MM-sucrose or MM-Avicel) at 25°C and 220 rpm unless otherwise indicated. Avicel PH 101 was obtained from Sigma-Aldrich (catalog no. 11365). For plate assays, *N. crassa* conidia were inoculated on 1.5% agar plates with Vogel's salts and 2% carbon source at 30°C for 24 hrs.

For microscopy, strains were inoculated in liquid MM for 16 hrs and washed with Vogel's salts. The resulting hyphae were inoculated on agarose only plates or with sucrose, CMC, or Avicel as the sole carbon source for an additional 5–6 hrs at 25°C. Just prior to imaging, 1 µg/ml of DAPI was added to the sample and incubated at room temperature for ∼15 min. A 0.5 cm×0.5 cm square of agarose with growing hyphae was used for imaging. Microscopy was performed on a Deltavision Spectris DV4 deconvolution microscope (Applied Precision Instruments). SVI Huygens Professional and Bitplane Imaris were used for image processing.

### Plasmid construction and transformation

Genomic DNA (gDNA) from FGSC 2489 using for template was extracted according to the method of Lee and Taylor (http://www.fgsc.net/fgn35/lee35.pdf). Two versions of *cre-1* plasmids were constructed according to Sun *et al.*, [79]. Briefly, plasmid pNeurA-8807 containing *cre-1* under the *ccg-1* promoter was constructed as follows: a DNA fragment corresponding to the *cre-1* open reading frame (ORF) was amplified by polymerase chain reaction (PCR) using WT gDNA as the template and primers 8807-AF and 8807-R (Table S7) which contain the LIC adapter. Plasmid pNeurD-8807 contains the *cre-1* ORF and 1 kbp of its upstream sequence, and was amplified using the primers 8807-DF and 8807-R (Table S7). The resulting PCR fragments were inserted into vectors as described in [79]. Plasmid inserts were sequenced by the UC Berkeley DNA Sequencing Facility.

One µg of plasmid DNA was transformed into a (*his-3*; Δ*cre-1 a*) strain as described [80]; constructs were targeted to the *his-3* locus by homologous recombination. Correct integration at the *his-3* locus in heterokaryotic transformants was confirmed by GFP fluorescence and PCR. To recover homokaryotic strains, His^+^ GFP^+^ transformants were crossed with a *his-3*; Δ*cre-1 A* strain. Progeny were selected for histidine prototrophy and GFP fluorescence, and screened for complementation of Δ*cre-1* by evaluating growth on Avicel and assessing cellulase activity. Complemented strains used in this study were termed Pn-*cre-1* (for regulation of the *cre-1* gene by the *cre-1* promoter) and Pc-*cre-1* (for regulation of the *cre-1* gene by the *ccg-1* promoter).

### Quantitative reverse transcription PCR

Total RNA was isolated using TRIzol (Invitrogen) according to the manufacturer's instruction and treated with DNase (Turbo DNA-free kit; Ambion) [81]. For quantitative real-time reverse transcription PCR (qRT-PCR), an EXPRESS one-step SYBR® GreenER™ with pre-mixed ROX kit (Invitrogen, catalog no. 11790-200) was used following the manufacturer's instructions, using an ABI 7300 real-time PCR system. Primers used for *cbh-1* (NCU07340), *cbh-2* (NCU09680; *gh6-2*), *gh5-1* (NCU00762), NCU03181, *cre-1* (NCU08807) and β-glucosidase (NCU04952; *gh3-4*) are shown in Table S7. Three replicates were performed per experiment. Experimental set up and data analyses were done as previously described [82]. Expression of the actin gene, *act-1* (NCU04173), was used as an endogenous control for all experiments.

### cDNA labeling and microarray analysis

Ten-day old conidia of WT (FGSC 2489) or Δ*cre-1* (FGSC 10372) strain were inoculated into Vogel's [78] liquid MM (2% sucrose) and grown for 16 hrs. At this time point, the biomass of these two samples was similar. Mycelia were centrifuged and washed with 1× Vogel's salts. Mycelia were then transferred into either Vogel's media with 2% sucrose or 2% Avicel and grown in constant light for 4 hrs. Mycelia were harvested by filtration and immediately frozen in liquid nitrogen. Total RNA was extracted as described above. ChipShot™ Indirect Labeling/Clean-Up System (Promega, catalog no. Z4000) and CyDye Post-Labeling Reactive Dye Pack (GE, catalog no. RPN5661) were used to synthesize and label cDNA according to the manufacturer's instruction, except that 10 µg of RNA was used. The Pronto! Hybridization Kit (Corning, catalog no. 40076) was used for microarray hybridization according to the manufacturer's specifications. A dye-swap was used to avoid the differential hybridization of each sample (Figure S2).

Data analyses were performed as previously described [83]. A GenePix 4000B scanner (Axon Instruments) was used to acquire images, and GenePix Pro6 software was used to quantify hybridization signals and collect the raw data. Normalized expression values were analyzed by using BAGEL (Bayesian Analysis of Gene Expression Levels) [84]. Genes showing a statistically significant difference in expression level between any two samples were identified using an in-house PERL script (available at http://glasslab.weebly.com/). Genes showing at least a 2-fold increase or decrease in relative expression level were used for Functional Category Analysis, as described in [43]. All profiling data are available at the MIAME-compliant databases (Filamentous Fungal Gene Expression Database; Experiment ID = 61; http://bioinfo.townsend.yale.edu/browse.jsp) and GEO (Accession Number = GSE30313; http://www.ncbi.nlm.nih.gov/geo/info/linking.html).

### Proteomics sample preparation and mass spectrometry

Total extracellular protein concentration was determined using a Bio-Rad Protein Assay kit II (Bio-Rad, catalog no. 500-0002). Twenty µl of unconcentrated culture supernatants were treated with 5× SDS loading dye (0.2% of β-mercaptoethanol was added before use) and boiled for 10 minutes before loading onto Criterion 4–15% Tris-HCl polyacrylamide gels (Bio-Rad). ProtoBlue Safe Colloidal Coomassie Blue G-250 was used for gel staining (National Diagnostics). For mass spectrometry analysis, the Δ*cre-1* strain was grown on 2% Avicel medium for 7 days. Cultures were centrifuged and the resulting supernatants were filtered through a 0.22 µm filter (Corning) and concentrated 10 times with 10 kDa MWCO PES spin concentrators (Millipore). About 2∼3 µg protein was used for trypsin digestion as described in [27]. Mass spectrometry was performed by the QB3/Chemistry Mass Spectrometry Facility at UC Berkeley as previously described [27]. The resulting data from LC-MS/MS analysis of trypsin-digested proteins were analyzed by using Protein- Lynx Global Server software (version 2.3, Waters). The processed data were searched against the *N. crassa* database (Broad Institute; http://www.broad.mit.edu/annotation/genome/neurospora/Home.html).

### Enzyme activity measurements

Endoglucanase activity in the culture supernatants was measured with an azo-CMC kit (Megazyme, Lot# SCMCL) according to manufacturer's instruction. Avicelase assays were performed as described in Tian *et. al.*, [27]. In brief, one volume of 7-day culture supernatant was mixed with one volume of substrate solution containing 5 mg/ml Avicel and 50 mM NaAc buffer, pH 5.0 at 37°C for 5 hrs with shaking. Then, glucose and cellobiose concentration were measured by coupled enzyme assays.

### Promoter analysis and chromatin immunoprecipitation PCR (ChIP-PCR)

Regions 1 kbp upstream of predicted translational start sites were downloaded from the Broad institute *N. crassa* database (http://www.broadinstitute.org/annotation/genome/neurospora/MultiDownloads.html), and the promoter regions of potential targets of CRE-1 were extracted from this list. The 5′-SYGGRG-3′ pattern was used for PASTER motif searching (cutoff score = 6). The Chi-square statistic was calculated by comparing the number of genes from a particular microarray dataset that contained the motif to the number of genes in the entire genome that contained the motif. P values were derived from corresponding Chi-square.

The ChIP protocol was modified from published procedures [85]. The Pc-*cre-1-gfp* strain was used for all ChIP experiments. Starting materials were identical to samples used for microarray analyses (above). A 16 hr culture grown in MM-sucrose was transferred to MM-Avicel for 4 hrs. Samples were fixed in 1% formaldehyde for 20 min and quenched by adding glycine to a final concentration of 0.125 M. Immunoprecipitation was performed using anti-GFP antibody (Roche, Cat. No. 1-814-460) or RNA Pol II antibody (Abcam, Cat. No. ab5095) as a positive control. Mouse IgG was used as negative control. After incubation overnight at 4°C, the bound antibody was extracted using Protein G bound Dynabeads (Invitrogen), washed in lysis buffer and resuspended in TES. Crosslinking from the eluted chromatin was removed by incubation at 65°C overnight followed by a Proteinase K and RNase A digestion. After DNA extraction, the pellets were re-suspended in 60 µL of TE. Afterwards, 1 µL of DNA solution was used for PCR. The primer pairs were constructed from the upstream 1 kbp of the ORF of putative CRE-1 targets which contained two closely spaced 5′-SYGGRG-3′ motifs (Tables S4, S5).

## Supporting Information

Figure S1 **Growth rate of wild type (FGSC 2489) and Δ** ***cre-1*** **(FGSC 10372) strains on different carbon sources (2%) in race tubes** [**1**] **at 25°C.** Agar only media (no added carbon source) was used as control. 1. Ryan F, Beadle G, Tatum E (1943) The tube method of measuring the growth rate of Neurospora. Am J Bot 30: 784–799.
(TIF)

Figure S2 **Experimental design for microarray analysis of wild type and Δ** ***cre-1*** **strains grown in either MM-sucrose or MM-Avicel cultures.** Samples were pre-grown in MM-sucrose for 16 hours, washed and centrifuged. Mycelia were then transferred into either minimal medium with 2% sucrose (MM) or minimal medium with 2% Avicel (Avi) as sole carbon sources and the culture was allowed to grow for another 4 hrs. A closed circuit design for microarray comparisons was used, which is statistically robust and improves resolution and precision [1]. Each arrow represents a hybridization. The arrowhead indicates a Cy5-labeled cDNA, while the opposite end represents Cy3-labeled cDNA. 1. Townsend JP, Taylor JW (2005) Designing experiments using spotted microarrays to detect gene regulation differences within and among species. Methods Enzymol 395: 597–617.
(TIF)

Figure S3 **Gene expression of a set of cellulases in a wild type strain (FGSC 2489).** A culture of FGSC 2489 was grown for 16 hours in MM-sucrose and subsequently transferred to MM-Avicel. RNA was extracted at different time points (noted above), and subjected to quantitative RT-PCR using primers to an intracellular β-glucosidase (NCU00130) or *gh5-1* (NCU00762) or *cbh-1* (NCU07340) or *gh61-4* (NCU07898) or *gh6-2* (NCU009682) (see Materials and Methods). Primer sequences are listed in Table S1. Expression was normalized to that of the *N. crassa* actin gene (NCU04173). Expression levels were verified by subsequent microarray analyses.
(TIF)

Figure S4 **Direct targets of CRE-1 confirmed by ChIP-PCR.** The P*c-cre-1-gfp* strain was used for ChIP DNA preparation (See Materials and Methods). ChIP-PCR was conducted to examine the individual selected potential targets of CRE-1. The negative control is a 332 bp region in the genome of *N. crassa* Supercontig 10.7 (2726–3057) that shows no expression under any conditions (unpublished results). Lanes 1–5 for the negative control (C-) above are: 1. DNA from GFP antibody pull-down assay; 2. DNA from Pol II antibody pull-down assay; 3. IgG pulldown; 4. Input DNA diluted as 1∶40; 5. H_2_O control. The positive control for ChIP-PCR used RNA Pol II antibody (abcam, Cat. No. ab5095) and primers to the promoter region of GAPDH (NCU01528), which is a constitutively expressed gene in *N. crassa* (unpublished observations) (see Table S4 for primer sequences). Lanes 1–5 represent PCR templates for the positive control (Pol II) above: 1. DNA from Pol II antibody pull-down assay; 2. DNA from IgG pull down; 3. DNA from beads only; 4. Input DNA diluted as 1∶40; 5. H_2_O control for PCR. For identifying direct target genes of CRE-1, ChIP-PCR was performed on multiple regions of the promoters from 16 selected putative targets; results from 13 are shown (gene ID above). Labelled lanes 1–5 represent: 1. DNA from the GFP antibody pull-down assay; 2. DNA from IgG pulldown; 3. DNA from beads only control; 4. Input DNA diluted as 1∶40; 5. H_2_O control for PCR. Of the 16 putative target genes, promoter regions for 9 of the genes were significantly enriched in by GFP antibody immunoprecipitation relative to all other lanes (Fig. 8). For sizing of PCR products, the 1 kbp Plus DNA Ladder from Invitrogen was used.
(TIF)

Table S1 (XLS)

Table S2 (XLS)

Table S3 (XLS)

Table S4 (XLS)

Table S5 (XLS)

Table S6 (XLS)

Table S7 (XLS)
