# Supplementary figures and images for: Identification of the CRE-1 Cellulolytic Regulon in Neurospora crassa
Source: PLoS One. 2011 Sep 29;6(9):e25654. doi: 10.1371/journal.pone.0025654 (PMC3183063; doi:10.1371/journal.pone.0025654)

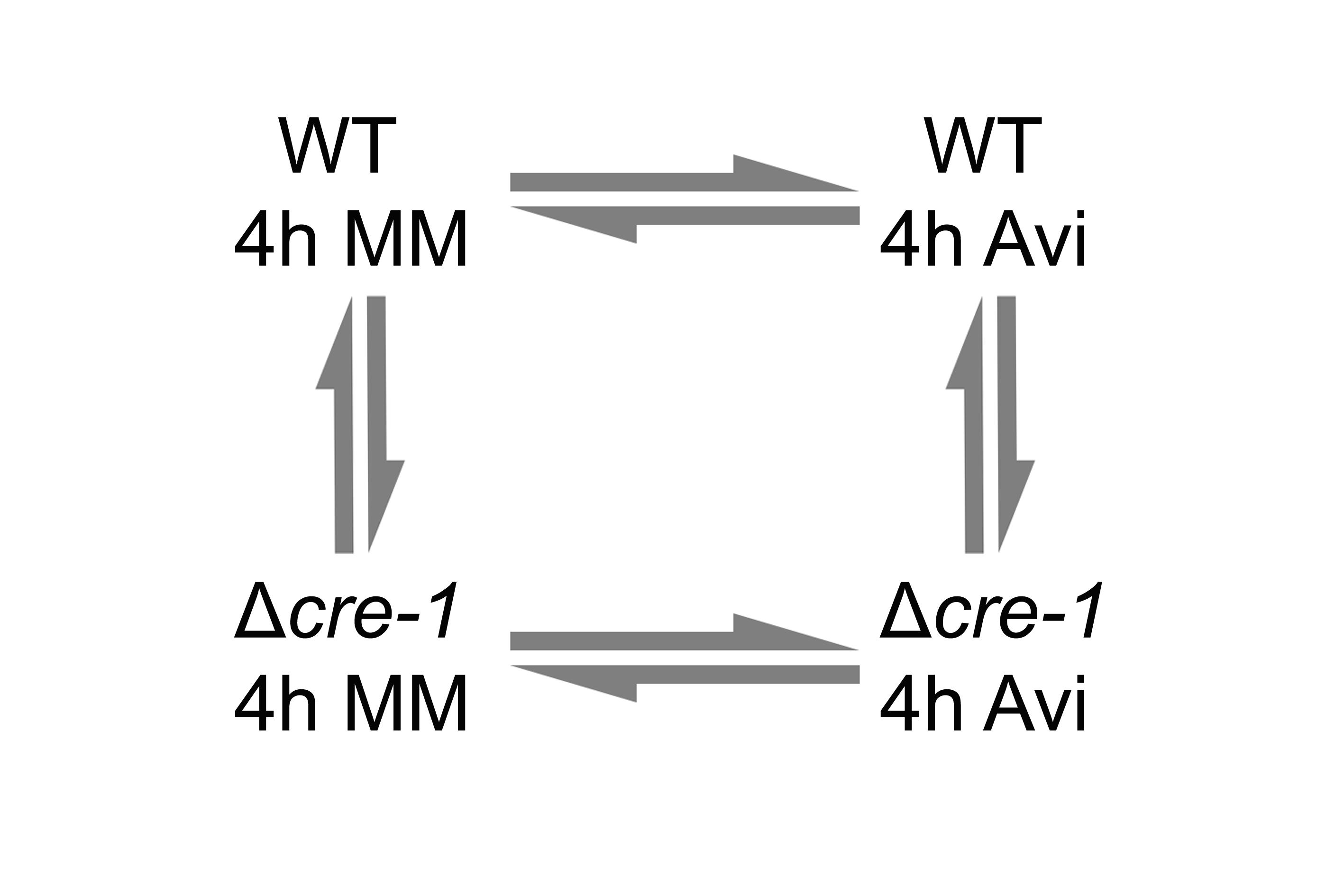

Supplement: Figure S1 — Growth rate of wild type (FGSC 2489) and Δ cre-1 (FGSC 10372) strains on different carbon sources (2%) in race tubes [1] at 25°C. Agar only media (no added carbon source) was used as control. 1. Ryan F, Beadle G, Tatum E (1943) The tube method of measuring the growth rate of Neurospora. Am J Bot 30: 784–799. (TIF) [file pone.0025654.s001.tif]

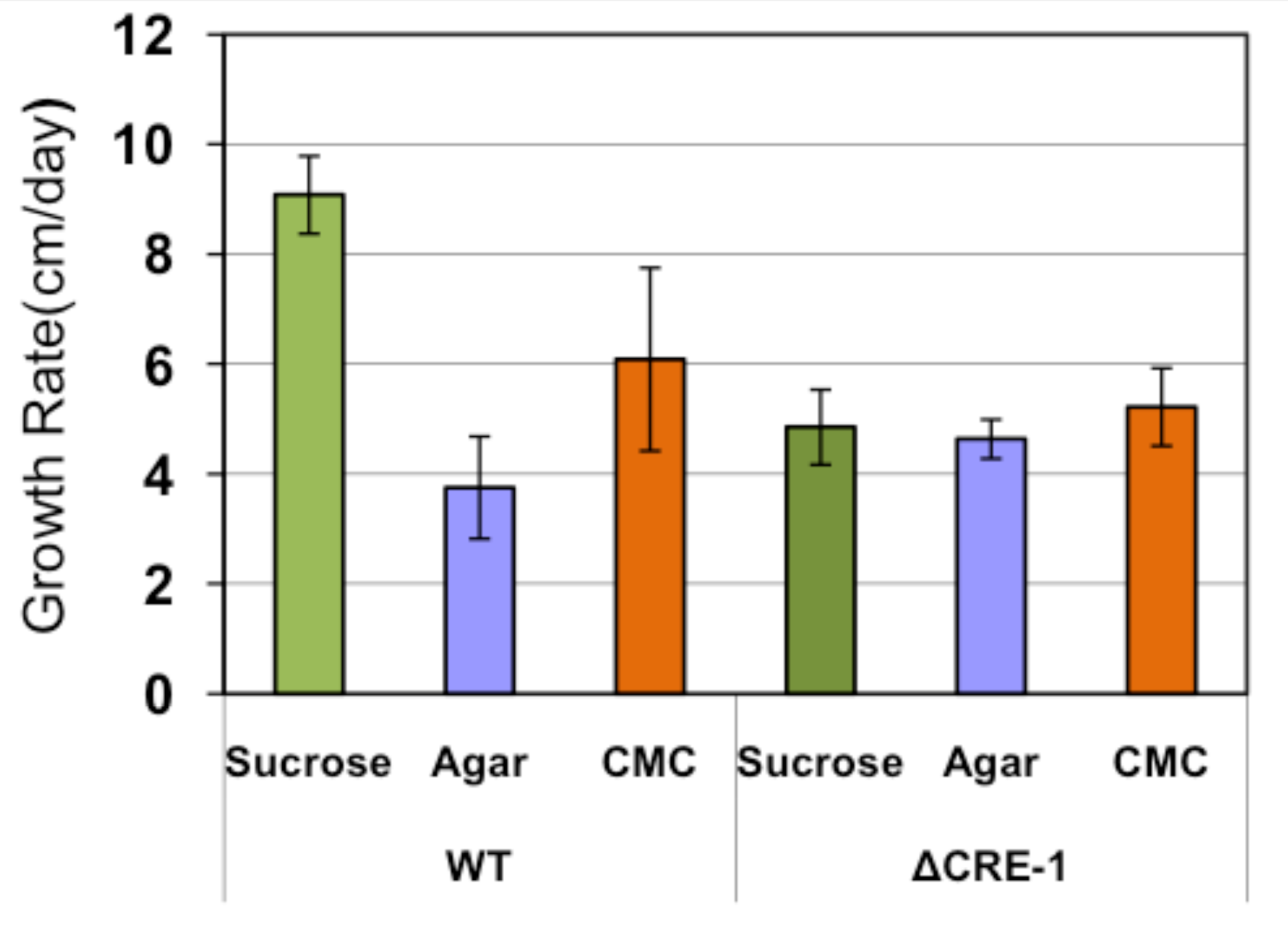

Supplement: Figure S2 — Experimental design for microarray analysis of wild type and Δ cre-1 strains grown in either MM-sucrose or MM-Avicel cultures. Samples were pre-grown in MM-sucrose for 16 hours, washed and centrifuged. Mycelia were then transferred into either minimal medium with 2% sucrose (MM) or minimal medium with 2% Avicel (Avi) as sole carbon sources and the culture was allowed to grow for another 4 hrs. A closed circuit design for microarray comparisons was used, which is statistically robust and improves resolution and precision [1]. Each arrow represents a hybridization. The arrowhead indicates a Cy5-labeled cDNA, while the opposite end represents Cy3-labeled cDNA. 1. Townsend JP, Taylor JW (2005) Designing experiments using spotted microarrays to detect gene regulation differences within and among species. Methods Enzymol 395: 597–617. (TIF) [file pone.0025654.s002.tif]

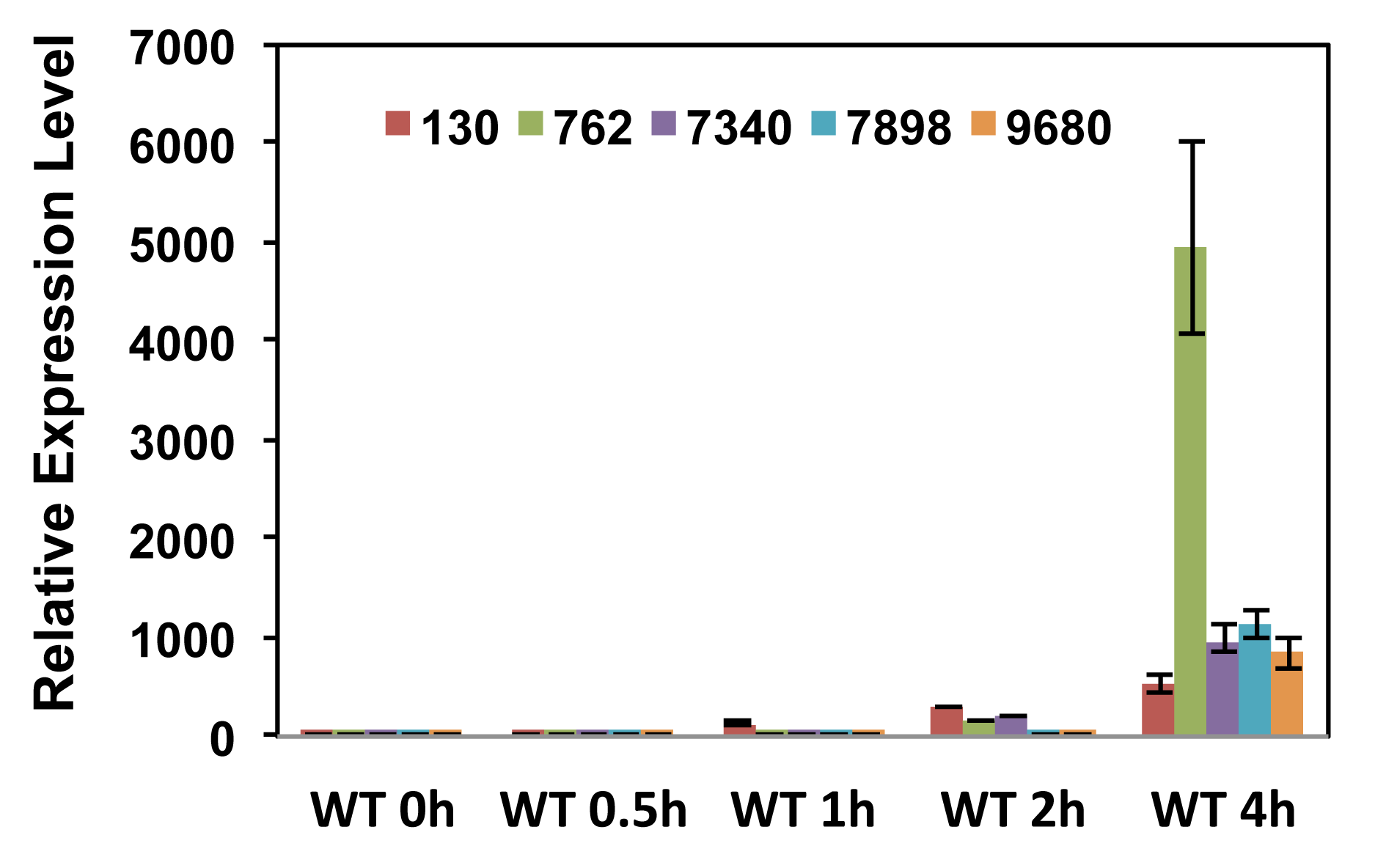

Supplement: Figure S3 — Gene expression of a set of cellulases in a wild type strain (FGSC 2489). A culture of FGSC 2489 was grown for 16 hours in MM-sucrose and subsequently transferred to MM-Avicel. RNA was extracted at different time points (noted above), and subjected to quantitative RT-PCR using primers to an intracellular β-glucosidase (NCU00130) or gh5-1 (NCU00762) or cbh-1 (NCU07340) or gh61-4 (NCU07898) or gh6-2 (NCU009682) (see Materials and Methods). Primer sequences are listed in Table S1. Expression was normalized to that of the N. crassa actin gene (NCU04173). Expression levels were verified by subsequent microarray analyses. (TIF) [file pone.0025654.s003.tif]

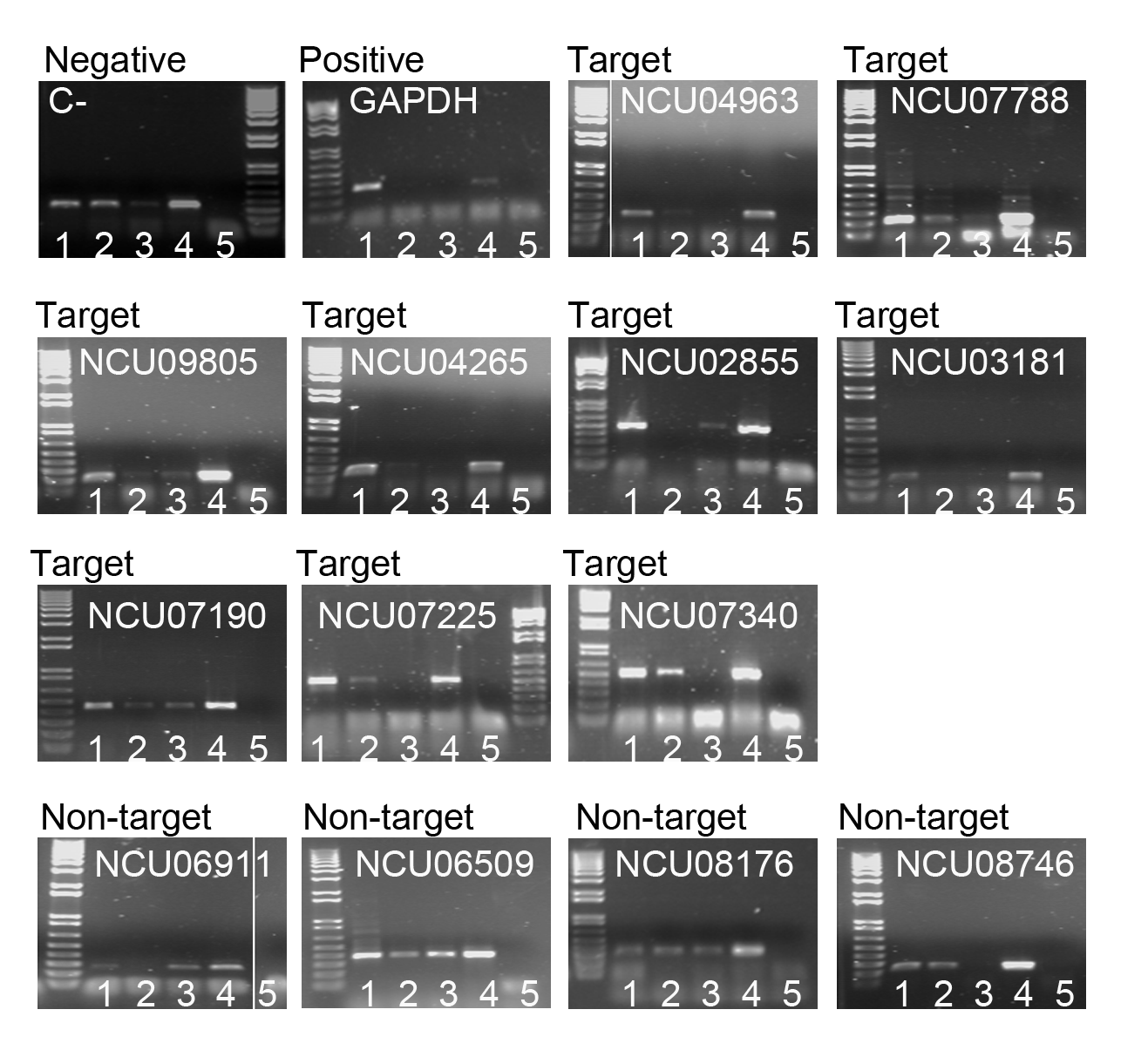

Supplement: Figure S4 — Direct targets of CRE-1 confirmed by ChIP-PCR. The Pc-cre-1-gfp strain was used for ChIP DNA preparation (See Materials and Methods). ChIP-PCR was conducted to examine the individual selected potential targets of CRE-1. The negative control is a 332 bp region in the genome of N. crassa Supercontig 10.7 (2726–3057) that shows no expression under any conditions (unpublished results). Lanes 1–5 for the negative control (C-) above are: 1. DNA from GFP antibody pull-down assay; 2. DNA from Pol II antibody pull-down assay; 3. IgG pulldown; 4. Input DNA diluted as 1∶40; 5. H2O control. The positive control for ChIP-PCR used RNA Pol II antibody (abcam, Cat. No. ab5095) and primers to the promoter region of GAPDH (NCU01528), which is a constitutively expressed gene in N. crassa (unpublished observations) (see Table S4 for primer sequences). Lanes 1–5 represent PCR templates for the positive control (Pol II) above: 1. DNA from Pol II antibody pull-down assay; 2. DNA from IgG pull down; 3. DNA from beads only; 4. Input DNA diluted as 1∶40; 5. H2O control for PCR. For identifying direct target genes of CRE-1, ChIP-PCR was performed on multiple regions of the promoters from 16 selected putative targets; results from 13 are shown (gene ID above). Labelled lanes 1–5 represent: 1. DNA from the GFP antibody pull-down assay; 2. DNA from IgG pulldown; 3. DNA from beads only control; 4. Input DNA diluted as 1∶40; 5. H2O control for PCR. Of the 16 putative target genes, promoter regions for 9 of the genes were significantly enriched in by GFP antibody immunoprecipitation relative to all other lanes (Fig. 8). For sizing of PCR products, the 1 kbp Plus DNA Ladder from Invitrogen was used. (TIF) [file pone.0025654.s004.tif]
